# Monitoring child health and development in the context of the SDGs: challenges and perspectives in early childhood

**DOI:** 10.1590/0034-7167-2025-0158

**Published:** 2026-10-26

**Authors:** Jeniffer Stephanie Marques Hilário, Letícia Pancieri, Rosane Meire Munhak Silva, Diene Monique Carlos, Maria de La Ó Ramallo Verissimo, Débora Falleiros de Mello

**Affiliations:** I Universidade de São Paulo. Ribeirão Preto, São Paulo, Brazil; II Universidade Estadual do Oeste do Paraná. Foz do Iguaçu, Paraná, Brazil; III Universidade de São Paulo. São Paulo, São Paulo, Brazil

**Keywords:** Child Health, Child Development, Primary Health Care, Sustainable Development Goals, Nursing., Salud Infantil, Desarrollo Infantil, Atención Primaria de Salud, Objetivos de Desarrollo Sostenible, Enfermería.

## Abstract

**Objectives:**

to analyze maternal perceptions regarding health monitoring and surveillance of child development in light of Sustainable Development Goals 3, 5, and 10.

**Methods:**

a qualitative study focusing on sustainable development for healthy early childhood. Twenty-seven maternal interviews were conducted in a small municipality in southeastern Brazil, with inductive data analysis.

**Results:**

interaction was identified in child monitoring, communication through messages, the advantages and disadvantages of a Child’s Health Record, information seeking, detailing of a child’s routine and parental practices. Weaknesses in communication were found during consultations and in child development assessment.

**Final Considerations:**

the strengths and weaknesses identified constitute challenges for the comprehensive care of children and for public policies, which align with and diverge from the Sustainable Development Goals studied. The implications for professional practice include the need for advances in clinical practice, expanded communication, and a responsive approach in early childhood.

## RESUMEN

**Objetivos:**

analizar las percepciones maternas sobre el monitoreo de la salud y la vigilancia del desarrollo infantil a la luz de los Objetivos de Desarrollo Sostenible 3, 5 y 10.

**Métodos:**

estudio cualitativo centrado en el desarrollo sostenible para una primera infancia saludable. Se realizaron 27 entrevistas maternas en un pequeño municipio del sureste de Brasil, mediante análisis inductivo de datos.

**Resultados:**

se identificó interacción en el seguimiento del niño, la comunicación mediante mensajes, las ventajas y desventajas de su historial médico, la búsqueda de información y el detalle de su rutina y las prácticas parentales. Se detectaron deficiencias en la comunicación durante las consultas y la evaluación del desarrollo infantil.

**Consideraciones Finales:**

las fortalezas y debilidades identificadas constituyen desafíos para la atención integral de la infancia y las políticas públicas, que se alinean y divergen con los Objetivos de Desarrollo Sostenible estudiados. Las implicaciones para la práctica profesional incluyen la necesidad de avances en la práctica clínica, una comunicación más amplia y un enfoque receptivo en la primera infancia.

## INTRODUCTION

Inequalities in early childhood and access to health and healthy child development, particularly in lowand middle-income countries, imply a constant movement towards achieving the Sustainable Development Goals (SDGs), in favor of inclusive, equitable, and quality opportunities^(1)^. Monitoring child development has shown weaknesses for a large proportion of children under 3 years of age^(2-4)^. Studies indicate that deprivations related to life, health, learning opportunities, and positive interactions have a greater impact on children living in precarious conditions^(5,6)^.

The 2030 Agenda for Sustainable Development is an action plan to eradicate poverty, reduce inequalities, protect the environment, and promote social and economic development^(7)^. This study emphasizes SDGs 3, 5, and 10, highlighting their unique relevance to monitoring child health and development.

SDG 3 focuses on ensuring healthy lives and promoting well-being at all ages^(7)^. For children and their families, SDG 3 is more directly linked to vital needs, with continuous monitoring of child growth and development being a fundamental component for prevention and promotion of childhood health^(8)^.

Child health and its connection to SDG 5 refer to promotion of shared responsibility and recognition of parental caregivers as the primary educators of their children, with parental education, adequate information, accessible public services, income transfer, and other factors being essential^(7)^.

SDG 10 involves ensuring equal opportunities and reducing inequalities, linked to the challenges of reducing socioeconomic disparities, and, in circumstances vulnerable to child development, includes poverty, low parental education, and child maltreatment, causing inequalities of opportunity and unfavorable outcomes throughout life^(9)^.

A redefinition of the purposes of global child health in the post-2015 era has been put forward so that children and adolescents are at the center of the SDGs^(10)^, highlighting the importance of equity, rights, and the need for the formulation and implementation of policies informed by scientific evidence. Health surveillance is highlighted as crucial for child health in early childhood, with emphasis on routine follow-up, avoiding low adherence to child health recommendations^(11)^. Furthermore, monitoring child development is important for detecting developmental problems, especially in early childhood^(12,13)^.

The SDGs highlighted here emphasize the relationship with access to Primary Health Care (PHC) and the importance of safe, effective, and qualified actions^(7,14)^. However, there are challenges in addressing gaps in access to healthcare^(9,15,16)^, coupled with the fact that children in their early years are at risk of not reaching their developmental potential, particularly in lowand middle-income countries^(17)^, such as Brazil. Nursing care also faces challenges in the important task of consolidating the SDGs^(18)^. Supporting positive parenting and a healthy care environment, which are essential in human development trajectories, are also challenges^(19,20)^. Given the above, the importance of continuous monitoring and surveillance of child development in PHC is highlighted, and it is important to understand parental caregivers’ perceptions in the context of SDGs 3, 5, and 10, including their challenges and perspectives in early childhood.

## OBJECTIVES

To analyze maternal perceptions regarding health monitoring and surveillance of child development in light of SDGs 3, 5, and 10.

## METHODS

### Ethical aspects

The research was approved by the Research Ethics Committee (Certificate of Submission for Ethical Consideration 68073823.5.0000.5393), in accordance with Resolution 466/2012. Informed Consent Form was obtained in writing from all individuals involved in the study.

The quotations were selected to provide a concise overview of the topics, and were anonymized using a letter and a number, with M for mothers (M1, ... M27) and C for children (C1... C27), followed by their age in months.

### Study design

A qualitative design was employed to describe and understand maternal experiences in relation to their perceptions of child health monitoring and development surveillance in light of the SDGs^(7)^. The qualitative approach is particularly well-suited for capturing the richness of intersubjective perceptions and meanings^(21,22)^. The study followed the COnsolidated criteria for REporting Qualitative research^(23)^.

### Study setting

The study was carried out in the municipality of Itaú de Minas, Minas Gerais, Brazil, which is small, with an estimated population of 16,286 inhabitants^(24)^, and has five health units, all of which are part of the Family Health Strategy (FHS).

### Data source

Twenty-seven mothers, residing in the catchment areas of the five FHSs in the municipality, participated in the study. Women over 18 years of age, who had low-risk pregnancies, registered and under follow-up at FHS units, with children up to 1 year of age, were included. Women deprived of liberty or hospitalized, who do not speak Portuguese, and with children with special healthcare needs were excluded. Losses were considered those not found after three attempts to conduct a home visit (HV).

The five FHS units had a universe of 114 women with children under 1 year of age. Considering the children who were receiving healthcare in child health at the five FHS units and a distribution among these units, 41 women were invited. There were three refusals to participate, and 11 could not be found in HV after three attempts. A list of mothers in relation to each FHS unit was organized with attention to the inclusion criteria, with a sequence to encompass different ages of children during the first year of life and invitations to mothers to participate in the study. The interruption of interviews was motivated by data characteristics, richness, and sufficiency^(25)^ on maternal perceptions regarding health monitoring and surveillance of child development relevant to the object of study. Concerning health units, the distribution was ten participants from FHS 1, five from FHS 2, five from FHS 3, four from FHS 4 and three from FHS 5. Thus, 27 mothers participated in the study.

### Data collection and organization

Data collection was carried out through individual, in-person interviews, audio-recorded, during HVs between May and July 2023. Initial contact was made with community health workers (CHWs) during HV, and the interview was subsequently scheduled. Children under 1 year of age born between May 2, 2022, and April 30, 2023, were included, encompassing children of different ages during data collection. Initially, two pilot interviews were conducted to test the developed interview guide, which was later improved in some questions. Therefore, these two interviews were disregarded due to the changes made to the guide. Each interview lasted approximately 50 minutes and was conducted by the first author. No field notes were taken.

The interviews were guided by the question: how is the monitoring of <child’s name>’s health going? A series of other questions followed, seeking to understand maternal perceptions about access to and connection with the health unit, adherence to well-child visits, and information about child health and development. During the interviews, the Child’s Health Record was reviewed to identify notes about child health, highlighting developmental milestones, and to discuss its use and interpretation. The recorded interviews were transcribed in full by the first author and organized into individual files.

### Data analysis

Data analysis was based on inductive thematic analysis, including initial familiarization, repeated readings of the empirical material, code generation, topic naming, and descriptive and qualitative interpretation^(26)^.

Data were organized into individual digital files in Microsoft Word^®^, which were then uploaded to Atlas.ti^®^ for data management and coding of the collected information. Data analysis yielded elements that were discussed among the authors, with validation of codes and subcodes, ensuring the study’s reliability.

Familiarization with the data occurred through reading the interview transcripts, conducted separately by two authors (Phase 1). For coding, the authors held meetings with in-depth discussions about data interpretations. Thus, codes were created from the data (Phase 2). Subsequently, there was the combination and creation of subcodes and the search for topics (Phase 3). Then, the topics were reviewed, defined, and named (Phase 4). The final stage involved writing up the results.

## RESULTS

### Descriptive characterization of mothers and children

All 27 parental caregivers were mothers; of these, 18 were between 18 and 29 years old, and nine were 30 years old or older. The ages of the 27 children were 2 months (n=1), 3 months (n=2), 4 months (n=2), 5 months (n=3), 6 months (n=4), 7 months (n=2), 8 months (n=4), 9 months (n=3), 10 months (n=2), and 11 months (n=4).

Concerning maternal education, four had incomplete elementary school, eight had incomplete high school, 14 had completed high school, and one had completed higher education. Concerning family income, according to the minimum wage (MW) of 2023 ($1,320.00 *reais*)^(27)^, four participants reported income up to one MW, 20 participants, from two to three MWs, two, from four to five MWs, and one, of six or more MWs. In this context, 15 participants reported having no financial difficulties, and 12 reported having some type of financial need. In relation to work outside the home, 21 mothers reported not having outside work and that fathers/partners had a job.

In relation to cohabitation with a partner, four mothers reported not living with the child’s father, and three considered themselves single mothers. Regarding childcare, 14 mothers reported paternal involvement, and 13 mothers mentioned fathers/partners’ lack of involvement. Support in daily care was reported by 25 mothers, with the participation of 20 grandparents, three older siblings (over 20 years old), an aunt, and a nanny.

The mothers of six children reported not possessing a Child’s Health Record. None of the 21 record books contained entries on developmental milestones; 11 mothers read aloud from the records; and seven reported no use of the record. Of the 11 mothers who used the record book for reading, three indicated that they did not understand the written information.

### Monitoring child health and development: maternal perspectives

Qualitative results were organized into the following topics: “Interaction in monitoring child health”; “Communication via messaging”; “Child’s Health Record: facilities and barriers”; “Searching for information online about child care”; “The importance of knowing children’s daily lives”.

In the “Interaction in monitoring child health” section, there are positive reports and others that generate some dissatisfaction with the type of care received during children’s follow-up. Mothers highlighted the importance of paying attention and talking to them during the appointments.

*The doctor asks about everything. I like her very much. She investigates, tries to find out if I’ve been sick, and clarifies doubts.* (M2 C2, 6 months)

*We have nothing to complain about or anything we should focus on, because both the nurse and the doctor there are very attentive.* (M3 C3, 10 months)

Follow-up visits to the health unit are seen as important to verify that care and health are progressing well, to resolve doubts, and to provide parental reassurance.

*I think he’s been closely monitoring it. He’s tracking weight, height, making sure everything is alright, and checking his diet. We worry too much, don’t we? With this monitoring, I feel more at ease knowing he’s on the right track.* (M12 C12, 9 months)

*My son has been getting very sick. I don’t know if it’s from him being out at night, or if it’s his diet, it’s impossible to tell.* (M23 C23, 6 months)

On the other hand, there was dissatisfaction with the conduct of the services, which were due to their speed and focus mainly on aspects of growth and nutrition.

*They didn’t really look at his little body. They didn’t check if there was anything wrong with him. They vaguely asked what he was eating, weighed him, measured him, measured his head, and that was it.* (M17 C17, 9 months)

*There’s a lack of patience during consultations. It seems like they want to do everything in a rush, weighing and measuring in a hurry.* (M23 C23, 6 months)

There is a contrast between monitoring child health according to the routine appointment schedule and when a child is ill or experiencing a complication. There is also mention of an initial appointment without subsequent scheduling for children’s follow-up.

*It’s been a while* [...] *it was this year, I think it was the day he had an allergic reaction, an allergic reaction to insect bites.* (M10 C10, 11 months)

*Only when she was 1 month old* [...] *she even went to have the newborn screening test done and that was it.* (M7 C7, 8 months)

This topic addresses aspects of health monitoring in child healthcare, highlighting the fragile access to care and the fragmentation of services, which create gaps in achieving SDG 3 on good health.

In the “Communication via messaging” section, contact through WhatsApp^®^ is seen as beneficial, generally with the participation of CHWs. The use of online contact is primarily for notices and reminders. Childhood vaccination and appointment dates were the topics mentioned by mothers in their interactions with CHWs.

*When needed, I send a quick message on WhatsApp* ^®^ *to the secretary, and she asks the head nurse if there’s an opening for an appointment, to confirm the appointment, and to check if the prescription is ready.* (M3 C3, 10 months)

*Every time, the health workers give me information about the vaccination, and when I need to ask a question, I get in touch.* (M12 C12, 9 months)

Communication between parental caregivers and health units was limited to a notification channel, failing to explore and reinforce healthcare issues and expanding to other types of interventions towards positive parenting and monitoring child development. Thus, it also reflects limited reach in enhancing SDGs 3 and 5.

In the “Child’s Health Record: advantages and disadvantages” section, there are perceptions that this document is seen both as a rich source of information and a barrier to understanding. There are reports that indicate reliable content focused on childcare.

*They are very easy to read and understand what is written. There are also informative drawings* [...] *it’s a great source of information*. (M24 C24, 8 months)

*Every hour, especially on the internet, there’s something different being said. It leaves us directionless, lost about where to go and what to do. I often get a lot of my information from the Child’s Health Record.* (M15 C15, 11 months)

However, understanding the information contained in the Child’s Health Record presents some difficulties and raises questions.

*Regarding the head and development part, I’m still confused.* (M3 C3, 10 months)

*There are some parts that confuse me a little, especially when they talk about development, because in consultations they never ask about this so-called developmental leaps.* (M5 C5, 11 months)

Reports from mothers who mentioned infrequent use of the booklet indicated low interest and a need for more dialogue about its content.

*I’ve browsed through it a few times, but I haven’t actually read it. It didn’t really grab my attention. I only like looking at its information.* (M26 C26, 4 months)

*I read it when she was born. I was very insecure about newborn care* [...] *it was very enlightening. But there’s still a lack of support, especially for first-time mothers.* (M6 C6, 4 months)

The topic of the Child’s Health Record reveals inaccurate professional communication, mainly related to child development, suggesting its limited use during consultations. These aspects show little integration of this tool into the practice of child healthcare in PHC, which could contribute to achieving good health in SDG 3, parental education for SDG 5, and promoting child development for SDG 10.

The “Searching for information online about child care” topic presents reports of people turning to the internet to obtain information regarding children’s needs.

*On Google^®^, on the search page, I search for information about behavior because I have a brother who is autistic, so I’m always interested in these topics. I also look a lot at general development, such as developmental leaps, and what behaviors she needs to exhibit.* (M9 C9, 5 months)

*I do a lot of research on the Baby Center^®^ app. I also follow doctors, but most of the time it’s the mobile app.* (M4 C4, 8 months)

*On social media, I like to follow pages of pediatricians and hospitals. I look for information about developmental leaps so we can see the signs the child is showing and what we can do to calm them down.* (M1 C1, 10 months)

Concerning the diverse searches, many of the topics are listed in the Child’s Health Record, but there is no connection to this tool, causing online content to generate more interest. These aspects are concerning because they attempt to fill knowledge gaps about child health and development, potentially containing misinformation and being far removed from good health, child development promotion, and appropriate parenting practices, thus falling short of achieving SDGs 3, 5, and 10.

In the “The importance of knowing children’s daily lives” section, the need to discuss the family’s circumstances and children’s routine in more detail is highlighted.

*Having knowledge of our daily lives can also help with problems, allowing us to see where improvements can be made, so that we all benefit.* (M3 C3, 10 months)

*Knowing is essential to help take good care of children, especially those under 1 year old, who are so fragile and need so much care and attention.* (M24 C24, 8 months)

*I think it’s important that they know what her day is like* [...]*, if I need to talk to her more, if she’s developing well.* (M18 C18, 9 months)

A more detailed account of children’s daily lives is highlighted as important for obtaining more information that promotes good childcare and strengthens positive parenting practices. In this regard, it is understood that maternal reports point to weaknesses in care, suggesting challenges to achieving SDGs 3, 5, and 10.

Based on the collected reports and their relationship with the SDGs, a set of strengths and weaknesses regarding health monitoring and surveillance of child development stand out, which are presented in Chart 1.

**Chart 1:** Aspects of monitoring child health and development from the perspective of Sustainable Development Goals 3, 5 and 10

Sustainable Development Goals	Monitoring children’s health and development	
	Strengths	Weaknesses
Sustainable Development Goal 3 Good health	- Ask questions and discuss various topics; - Be a very attentive professional; - Follow up to identify the right paths; - Possess appropriate conduct; - Use the Child’s Health Record as a source of information; - Measure and assess at every consultation; - Schedule consultations as needed.	- Access difficulties; - Slow consultation times; - Insufficient clinical assessment; - Few observations; - Failure to meet the minimum consultation schedule; - Lack of recurring home visits; - Lack of verification of the sources accessed to obtain children’s information.
Sustainable Development Goal 5 Women’s empowerment	- Obtain information during consultations; - Maintain frequent contact with a community health worker via WhatsApp^®^; - Actively seek information about children’s health; - Use the Child’s Health Record to obtain information; - Possess a well-illustrated Child’s Health Record with children’s data; - Be familiar with children’s routine.	- Restricted questioning; - Lack of patience; - Unrestricted search for information on electronic devices; - Infrequent reading of the Child’s Health Record; - Little support for first-time mothers; - Communication via messaging limited to warnings and reminders.
Sustainable Development Goal 10 Equality in promoting child development	- Understand family problems and challenges; - Identify what can be improved; - Describe necessary stimuli and play activities; - Recognize the benefits of attentive and responsive care for children.	- Lack of clarity regarding child development during consultations; - Difficulties in understanding developmental milestones; - Little appreciation for children’s routine and family circumstances.

## DISCUSSION

The results provide significant insights into monitoring children’s health during well-child visits in PHC, suggesting a need to improve communication with parental caregivers, and child development assessment and monitoring. In this context, the importance of advancing clinical practice, expanding communication with families, and adopting a positive and responsive approach by healthcare professionals to promote a healthy early childhood and achieve SDGs 3, 5, and 10 is highlighted.

Maternal reports highlighted a need to establish dialogue, ask questions, clarify doubts, observe, and conduct more comprehensive clinical examinations. Some reports indicated that child development is rarely discussed during consultations, suggesting an invisibility of this aspect of care, hindering continuous child monitoring. These are areas that need improvement to foster positive interaction and expand parental interest, which can provide self-confidence and commitment to monitoring children’s health. These qualitative elements are intertwined with SDGs 3 and 5, as they address good child health and expanding opportunities for maternal empowerment towards comprehensive, equitable, and accessible care.

Scientific literature shows that continuous health monitoring and surveillance of child development yield important results in assessing child development and providing parental knowledge, helping to identify risks to child health^(28)^. Continuous monitoring also contributes to the prevention of unintentional injuries and to the screening for language delay and autism spectrum disorder^(29)^.

In the present study, maternal perceptions indicate a lack of guidance on stimulating child development, organizing care routines, and providing parenting practices by the primary healthcare team, whose focus appears to be on children’s physical and physiological health. The study suggests that families with young children need in-depth approaches to the quality of responsive parenting practices^(30)^. Other studies highlight the importance of dialogue with families in supporting the healthy growth and development of children, serving as a powerful mechanism to encourage action and accountability for child development at the family and community levels^(8,31)^.

Access to information via the Child’s Health Record drew attention because some mothers mentioned not being able to understand the information contained therein. These aspects suggest challenges to health literacy and, within the scope of this study, are mainly related to difficulties in understanding the milestones of child development and uncertainties about whether home practices are on the right track in providing care to children. Scientific studies indicate that, in the Brazilian context, there has been insufficient distribution of the Child’s Health Record, little guidance from professionals to families, little family participation in the use of this tool, and insufficient and irregular records of child development surveillance^(32)^. Therefore, these are important results for SDGs 3, 5, and 10, in their specific aspects related to good health, parental education, and the promotion of the full development of children.

Searches on electronic devices, using mobile applications and online social networks, are consistent with contemporary times because of the widespread use of the Internet, suggesting a need to acquire knowledge to meet demands and content on how to care for children. On the other hand, the search for information in other sources suggests a deficiency, pointing to a challenge in strengthening safe parenting and maternal empowerment. Studies indicate that it is of utmost importance that the digital information increasingly accessed by parental caregivers be accurate, understandable, and address their concerns^(33,34)^. The aspects presented are particularly related to SDG 5, considering the relevance of guaranteeing parental education and information pertinent to comprehensive healthcare. Therefore, qualified access to healthcare services and health education is fundamental for achieving better results in PHC^(18,35)^.

The results also provide insights into children’s routine, with maternal perceptions indicating the importance of carefully understanding daily care, allowing for intervention and adjustments to practices to prevent potential obstacles to growth and development. The process of understanding children’s daily lives and identifying the dilemmas faced by parental caregivers emerges as a unique value of care. This aspect is corroborated by the scientific literature, which highlights the relevance of paying close attention to socioeconomic patterns, family characteristics, caregivers, and parenting practices to mitigate risks to child development^(36)^. Failure to reach human development potential contributes to global cycles of poverty, inequality, and social exclusion^(22)^.

Recognizing daily realities highlights the need to pursue, through SDG 10, the reduction of inequalities of opportunity and unfavorable outcomes for children. Therefore, it is fundamental to promote a rich and critical awareness among parental caregivers, i.e., healthcare professionals must develop a high capacity for perceiving the care routine in different circumstances. Researchers propose the implementation of a daily log for data collection that emphasizes situations throughout the day with more details about feeding, sleep, play, parental interaction, frequency of daycare visits, social difficulties, among others, during the morning, afternoon, and evening periods^(37)^. This involves obtaining a clearer understanding of children’s routine in order to identify vulnerabilities and gain a deeper understanding of children’s needs within the family context^(37)^.

Studies indicate that primary child healthcare represents an opportunity to know children and their families in their context, identifying adverse conditions that compromise their health^(38)^ and intervening in the first 1,000 days of life, a crucial period for improving child health^(39)^. Furthermore, they highlight the importance of actions that combine the simultaneous monitoring of development and growth, considering the context of poverty situations, which can create barriers to relational health and everyday parenting practices^(40)^.

Childcare arrangements between parental caregivers and PHC services are proving increasingly important. However, many challenges remain in achieving the SDGs, given widespread global problems^(41,42)^. Therefore, there are challenges to investing in education for a positive impact on the lives of women, families, their communities, and, more generally, on society as a whole, highlighting the relationship between gender and health, and adding collaborative actions to the SDGs^(43)^. Brazilian experts highlight the need for commitment to poverty reduction, universalization of PHC and health literacy of the population^(44)^ as measures to promote inclusive development^(45)^. Health inequalities between Brazilian states and regions negatively affect the population, which may contribute to Brazil not achieving the SDG targets by 2030^(46)^.

### Study limitations

The study’s limitations relate to conducting interviews only with mothers and focusing on children’s first year of life, centered on the specificities of a small municipality. However, the relevance of the results and their analysis based on the studied SDGs is highlighted, portraying the potential and vulnerabilities of child health monitoring and child development surveillance, suggesting ways to perceive and conduct healthcare in PHC, which may be applicable in the context of FHS units, due to their guidelines of proximity to families and territorial action. Further investigations are important regarding the perceptions of other parental and non-parental caregivers, other age groups, and childhood adversities.

### Contributions to nursing and health

In primary child healthcare, nursing and other healthcare professionals face challenges in improving and enhancing the monitoring of child health and development related to SDGs 3, 5, and 10. Therefore, efforts are needed to increase interaction with mothers and parental caregivers in monitoring child health, deepen communication through messaging, expand the use of the Child’s Health Record, understand information searches from other sources to avoid misinformation, and improve data collection on children’s and their families’ daily lives. Thus, the need for greater professional responsiveness to improve child care and monitoring of child development in PHC is highlighted, in order to contribute more significantly to the SDGs emphasized here.

## FINAL CONSIDERATIONS

Maternal perceptions reveal strengths and weaknesses regarding health monitoring and surveillance of child development, highlighting challenges for comprehensive child care and public policies. Results involving good interaction and effective communication with mothers, provision of adequate information, and understanding of families’ daily lives align with the SDGs studied. On the other hand, the speed of care, insufficient clinical assessment, weak communication, low use of the Child’s Health Record, unrestricted use of online information, and little appreciation for children’s and families’ routines and daily lives suggest a disconnect from the analyzed SDGs.

The implications for professional practice relate to the need for advancements in clinical practice, enhanced communication, and a responsive approach in early childhood. The perspectives of SDGs 3, 5, and 10 are important for enhancing the longitudinal and comprehensive follow-up of children in PHC, strengthening continuity of care, reinforcing the urgency of improving communication and obtaining reliable information, and addressing daily demands, particularly for effective developmental monitoring from an early age.

## Data Availability

The research data are available within the article.
