# Supplementary material for: Monitoring child health and development in the context of the SDGs: challenges and perspectives in early childhood
Source: Rev Bras Enferm. 2026 Oct 26;79:e20250158. doi: 10.1590/0034-7167-2025-0158 (PMC13719132; doi:10.1590/0034-7167-2025-0158)
Supplement: Translation

# Translation: Acompanhamento da saúde e desenvolvimento infantil no contexto dos ODS: desafios e perspectivas na primeira infância

**DOI:** 10.1590/0034-7167-2025-0158pt

**Authors:** Jeniffer Stephanie Marques Hilário, Letícia Pancieri, Rosane Meire Munhak Silva, Diene Monique Carlos, Maria de La Ó Ramallo Verissimo, Débora Falleiros de Mello

**Affiliations:** I Universidade de São Paulo. Ribeirão Preto, São Paulo, Brasil; II Universidade Estadual do Oeste do Paraná. Foz do Iguaçu, Paraná, Brasil; III Universidade de São Paulo. São Paulo, São Paulo, Brasil

**Keywords:** Saúde da Criança, Desenvolvimento Infantil, Atenção Primária à, Saúde, Objetivos de Desenvolvimento Sustentável, Enfermagem.

## Abstract

**Objetivos:**

analisar as percepções maternas sobre acompanhamento da saúde e vigilância do desenvolvimento infantil à luz dos Objetivos de Desenvolvimento Sustentável 3, 5 e 10.

**Métodos:**

estudo qualitativo, com enfoque no desenvolvimento sustentável para primeira infância saudável. Foram realizadas 27 entrevistas maternas em um município de pequeno porte do sudeste brasileiro, com análise indutiva dos dados.

**Resultados:**

identificaram-se interação no acompanhamento da criança, comunicação por mensagens, facilidades e barreiras da Caderneta da Criança, busca de informações, detalhamento da rotina da criança e das práticas parentais. Houve fragilidades na comunicação durante consultas e na avaliação do desenvolvimento infantil.

**Considerações Finais:**

fortalezas e fragilidades apreendidas constituem desafios para o cuidado integral das crianças e para as políticas públicas, que se alinham e se distanciam dos Objetivos de Desenvolvimento Sustentável estudados. As implicações para a prática profissional incluem a necessidade de avanços na prática clínica, comunicação ampliada e abordagem responsiva na primeira infância.

## INTRODUÇÃO

As desigualdades na primeira infância e o acesso à saúde e ao desenvolvimento infantil saudável, principalmente em países de baixa e média renda, implicam um movimento constante para alcançar os Objetivos de Desenvolvimento Sustentável (ODS), em prol das oportunidades inclusivas, equitativas e de qualidade^(1)^. A vigilância do desenvolvimento infantil vem apresentando fragilidades para grande parte das crianças menores de 3 anos de idade^(2-4)^. Estudos apontam que privações relacionadas à vida, saúde, oportunidades de aprendizagem e boas interações têm maior peso para crianças em condições precárias de vida^(5,6)^.

A Agenda 2030 para o Desenvolvimento Sustentável é um plano de ação para erradicar a pobreza, reduzir as desigualdades, proteger o ambiente, e promover o desenvolvimento social e econômico^(7)^. O presente estudo enfatiza os ODS 3, 5 e 10, ressaltando suas singularidades para o acompanhamento da saúde e do desenvolvimento infantil.

O ODS 3 se volta à garantia de vidas saudáveis e promoção do bem-estar em todas as idades^(7)^. Para as crianças e suas famílias, o ODS 3 é mais diretamente vinculado às necessidades vitais, tendo relevância o acompanhamento contínuo do crescimento e do desenvolvimento infantil como um componente fundamental para prevenção e promoção da saúde na infância^(8)^.

A saúde da criança e sua articulação ao ODS 5 se referem à promoção da responsabilidade compartilhada e reconhecimento dos cuidadores parentais como os principais educadores dos seus filhos, sendo essenciais educação parental, informação adequada, serviços públicos acessíveis, transferência de renda, entre outros^(7)^.

O ODS 10 envolve a garantia da igualdade de oportunidades e redução das desigualdades, ligado aos desafios para redução das disparidades socioeconômicas, e, em circunstâncias vulneráveis ao desenvolvimento infantil, inclui a pobreza, a baixa escolaridade parental e os maus-tratos às crianças, ocasionando desigualdades de oportunidades e desfechos desfavoráveis ao longo da vida^(9)^.

Uma redefinição dos propósitos da saúde infantil global na era pós-2015 tem sido colocada para que crianças e adolescentes estejam no centro dos ODS^(10)^, ressaltando a importância da equidade, dos direitos e da necessidade da formulação e implementação de políticas informadas por evidências científicas. A vigilância em saúde é destacada como crucial para a saúde de crianças na primeira infância, sendo enfatizado o acompanhamento de rotina, evitando a baixa adesão às recomendações de saúde infantil^(11)^. Ainda, a vigilância do desenvolvimento infantil é importante para a detecção de problemas de desenvolvimento principalmente na primeira infância^(12,13)^.

Os ODS aqui destacados enfatizam a relação com o acesso à Atenção Primária à Saúde (APS) e a importância de ações seguras, eficazes e qualificadas^(7,14)^. No entanto, há desafios para suprir as lacunas no acesso aos cuidados de saúde^(9,15,16)^, somado ao fato de as crianças nos primeiros anos de vida correrem riscos de não atingir o seu potencial de desenvolvimento, principalmente em países de baixa e média renda^(17)^, como o Brasil. Para o cuidado de enfermagem, também há desafios para a relevante tarefa na consolidação dos ODS^(18)^. Também são desafios o apoio à parentalidade positiva e o ambiente de cuidado saudável, que são essenciais nas trajetórias de desenvolvimento humano^(19,20)^. Diante do exposto, ressaltam-se a relevância do acompanhamento contínuo e vigilância do desenvolvimento infantil na APS, sendo importante apreender as percepções de cuidadores parentais no contexto dos ODS 3, 5 e 10 com seus desafios e perspectivas na primeira infância.

## OBJETIVOS

Analisar percepções maternas sobre o acompanhamento da saúde e vigilância do desenvolvimento infantil à luz dos ODS 3, 5 e 10.

## MÉTODOS

### Aspectos éticos

A pesquisa foi aprovada em Comitê de Ética em Pesquisa (Certificado de Apresentação para Apreciação Ética n^o^ 68073823.5.0000.5393), de acordo com a Resolução nº 466/2012. O Termo de Consentimento Livre e Esclarecido foi obtido de todos os indivíduos envolvidos no estudo por meio escrito.

As citações foram selecionadas para fornecer um panorama conciso dos temas, sendo anonimizadas utilizando uma letra e um número, sendo M para mães (M1, ... M27) e C para crianças (C1... C27), seguidas pela sua idade em meses.

### Tipo de estudo

Um delineamento qualitativo foi empregado para descrever e compreender as experiências maternas em relação às suas percepções sobre o acompanhamento da saúde e vigilância do desenvolvimento da criança à luz dos ODS^(7)^. A abordagem qualitativa é particularmente adequada para capturar a riqueza de percepções e significados intersubjetivos^(21,22)^. O estudo seguiu o *COnsolidated criteria for REporting Qualitative research* ^(23)^.

### Cenário do estudo

O estudo foi realizado no município de Itaú de Minas, Minas Gerais, Brasil, que é de pequeno porte, com população estimada de 16.286 habitantes^(24)^, e conta com cinco unidades de saúde, sendo todas com Estratégia Saúde da Família (ESF).

### Fonte dos dados

Participaram do estudo 27 mães, residentes nas áreas de abrangência das cinco ESFs do município. Mulheres acima de 18 anos de idade, que tiveram gestação de risco habitual, cadastradas e em seguimento nas unidades de ESF, com crianças até 1 ano de idade, foram incluídas. Mulheres em privação de liberdade ou em internação hospitalar, que não falam em português e com criança com necessidades de atenção especial de saúde foram excluídas. Foram consideradas perdas aquelas não encontradas após três tentativas de realização da visita domiciliar (VD).

As cinco unidades de ESF tinham um universo de 114 mulheres com crianças menores de 1 ano de idade. Considerando as crianças que realizavam seguimento da saúde em puericultura nas cinco ESFs e uma distribuição entre essas unidades, foram convidadas 41 mulheres. Houve três recusas para participação, e 11 não foram encontradas em VD após três tentativas. Uma listagem das mães em relação a cada unidade de ESF foi organizada com atenção aos critérios de inclusão, com uma sequência para abarcar diferentes idades das crianças durante o primeiro ano de vida e a realização de convites às mães para participar do estudo. A interrupção das entrevistas foi motivada pelas características, riqueza e suficiência dos dados^(25)^ sobre as percepções maternas em relação ao acompanhamento da saúde e vigilância do desenvolvimento infantil pertinentes ao objeto de estudo. Em relação às unidades de saúde, a distribuição foi de dez participantes da ESF 1, sendo cinco da ESF 2, cinco da ESF 3, quatro da ESF 4 e três da ESF 5. Assim, participaram do estudo 27 mães.

### Coleta e organização dos dados

A coleta de dados foi realizada por meio de entrevistas individuais, presenciais e gravadas em áudio, durante VD, entre maio e julho de 2023. Houve um contato inicial juntamente com agentes comunitários de saúde (ACSs) em VD e, posteriormente, agendada a entrevista. Foram incluídas as crianças menores de 1 ano de idade nascidas entre 02/05/2022 e 30/04/2023, incluindo crianças em diferentes meses de idade na coleta. Preliminarmente, foram realizadas duas entrevistas como piloto para testar o roteiro elaborado, o qual foi aprimorado em algumas perguntas, sendo o motivo dessas duas entrevistas serem desconsideradas pelas mudanças realizadas no roteiro. Cada entrevista teve a duração média de 50 minutos, conduzidas pela primeira autora. Notas de campo não foram realizadas.

As entrevistas tiveram como pergunta norteadora: como está sendo o acompanhamento da saúde da/o <nome da criança>? Houve um encadeamento de outras perguntas buscando dialogar e apreender percepções maternas sobre o acesso e vínculo com a unidade de saúde, adesão às consultas de puericultura, e informações sobre a saúde e o desenvolvimento infantil. Durante as entrevistas, foi observada a Caderneta da Criança, visando identificar anotações contidas sobre a saúde da criança, com destaque para os marcos do desenvolvimento infantil e dialogar sobre o uso e leitura da mesma. As entrevistas gravadas foram transcritas na íntegra pela primeira autora e organizadas em arquivos individuais.

### Análise dos dados

A análise de dados foi pautada na análise temática indutiva, incluindo a familiarização inicial, leituras repetitivas do material empírico, geração de códigos, denominação de temas e interpretação descritiva e qualitativa^(26)^.

Os dados foram organizados em arquivos digitais individuais no programa *Microsoft Word* ^®^, que foram lançados no *software Atlas.t*i^®^ para gerenciamento dos dados e codificação das informações coletadas. A análise de dados trouxe elementos que foram discutidos entre as autoras, com validação dos códigos e subcódigos, garantindo a confiabilidade do estudo.

A familiarização com os dados ocorreu por meio da leitura das transcrições das entrevistas, realizada por duas autoras separadamente (Fase 1). Para a codificação, foram realizadas reuniões das autoras com discussões aprofundadas sobre as interpretações dos dados. Assim, foram criados códigos a partir dos dados (Fase 2). A seguir, houve a combinação e criação de subcódigos e a busca de temas (Fase 3). Em seguida, os temas foram revisados, definidos e nomeados (Fase 4). A etapa final envolveu a redação dos resultados.

## RESULTADOS

### Caracterização descritiva das mães e crianças

Todas as 27 cuidadoras parentais eram mães; dessas, 18 tinham 18 e 29 anos, e nove tinham 30 anos ou mais de idade. A idade das 27 crianças foi 2 meses (n=1), 3 meses (n=2), 4 meses (n=2), 5 meses (n=3), 6 meses (n=4), 7 meses (n=2), 8 meses (n=4), 9 meses (n=3), 10 meses (n=2) e 11 meses (n=4).

Quanto à escolaridade materna, quatro tinham ensino fundamental incompleto, oito, ensino médio incompleto, 14, ensino médio completo, e uma, ensino universitário completo. No tocante à renda familiar, de acordo com o salário mínimo (SM) do ano de 2023 (R$1.320,00)^(27)^, quatro participantes informaram renda até um SM, 20 participantes, de dois a três SMs, dois, de quatro a cinco SMs, e uma, de seis ou mais SMs. Neste contexto, 15 participantes relataram não ter dificuldades financeiras, e 12 referiram ter algum tipo de necessidade financeira. Em relação ao trabalho fora de casa, 21 mães informaram não ter trabalho externo e que os pais/companheiros tinham um trabalho.

Quanto à convivência com companheiro, quatro mães informaram não estar residindo com o pai da criança, e três consideraram-se mães solo. Em relação aos cuidados da criança, 14 mães relataram participação paterna, e 13 mães mencionaram a não participação do pai/companheiro. O apoio no cuidado cotidiano foi referido por 25 mães, com a participação de 20 avós da criança, três irmãos mais velhos (acima de 20 anos), uma tia e uma babá.

As mães de seis crianças referiram não possuir a Caderneta da Criança. Em nenhuma das 21 cadernetas foram identificadas anotações sobre os marcos do desenvolvimento infantil; 11 realizavam leituras; e sete não relataram qualquer uso da mesma. Das 11 mães que utilizaram para leitura, três apontaram que não compreendiam as informações escritas.

### Acompanhamento da saúde e vigilância do desenvolvimento da criança: percepções maternas

Os resultados qualitativos foram organizados nos seguintes temas: “Interação no acompanhamento da saúde infantil”; “Comunicação por mensagens”; “Caderneta da Criança: facilidades e barreiras”; “Busca de informações *online* sobre o cuidado da criança”;” A importância de conhecer o cotidiano da criança”.

No tema “Interação no acompanhamento da saúde infantil”, há relatos positivos e outros que geram certa insatisfação com o tipo de atendimento no acompanhamento da criança. As mães destacaram que é importante dar atenção e conversar com elas durante as consultas.

*A doutora pergunta sobre tudo. Eu gosto muito dela. Ela investiga, procura saber se teve doente, esclarece dúvidas.* (M2 C2, 6 meses)

*A gente não tem nada para reclamar ou algo que deveria focar, porque ali tanto a enfermeira quanto a doutora são bem atentas.* (M3 C3, 10 meses)

O acompanhamento na unidade de saúde é visto como relevante para averiguar se os cuidados e a saúde estão caminhando bem, para resolução de dúvidas e segurança parental.

*Eu acho que vem acompanhando de perto. Acompanha peso, altura, se está tudo certo, como está a alimentação. A gente preocupa demais, né? Com esse acompanhamento, fico mais tranquila para ver se está indo pelo caminho certo.* (M12 C12, 9 meses)

*Meu menino anda ficando muito doente. Não sei se é ele ficar de noite na rua, se é a alimentação, não dá para saber.* (M23 C23, 6 meses)

Por outro lado, houve insatisfação sobre as condutas nos atendimentos pela rapidez e centrados, principalmente, em aspectos do crescimento e alimentação.

*Eles não olharam direito o corpinho dele. Ver se tem algo errado com ele. Perguntam por alto o que ele está comendo, pesa, mede, mede a cabeça e só.* (M17 C17, 9 meses)

*Falta um pouco mais de paciência durante as consultas. Parece que quer fazer tudo correndo, pesa e mede correndo.* (M23 C23, 6 meses)

Há uma contraposição entre o acompanhamento da saúde da criança no calendário de atendimentos de rotina e quando a criança está doente ou apresenta alguma intercorrência. Há também menção ao atendimento inicial sem agendamentos posteriores para o seguimento da criança.

*Faz um tempinho* [...] *foi esse ano ainda, acho que foi o dia que deu alergia nele, alergia à picada de inseto.* (M10 C10, 11 meses)

*Somente quando ela tinha 1 mês de vida* [...] *foi até para fazer o teste do pezinho e só.* (M7 C7, 8 meses)

Este tema traz aspectos sobre o acompanhamento da saúde na puericultura com apontamentos sobre o acesso frágil ao cuidado e a fragmentação assistencial, com lacunas para atingir o ODS 3 sobre a boa saúde.

No tema “Comunicação por mensagens”, o contato por meio do *WhatsApp* ^®^ é visto como benéfico, em geral com a participação de ACS. A utilização do contato *online* é voltada para avisos e lembretes. A vacinação infantil e datas de atendimentos foram os tópicos citados pelas mães na interlocução com os ACSs.

*Quando preciso, eu mando alguma coisinha no WhatsApp^®^ para a secretária, e ela pergunta para a enfermeira responsável se tem encaixe de consulta, para confirmar a consulta, se a receita ficou pronta.* (M3 C3, 10 meses)

*Toda vez, as agentes de saúde avisam sobre a vacinação, e quando eu preciso tirar alguma dúvida, eu entro em contato.* (M12 C12, 9 meses)

A comunicação entre os cuidadores parentais e as unidades de saúde ficou restrita a um canal de avisos, deixando de explorar e reforçar temas sobre cuidados com a saúde e de se expandir para outros tipos de intervenção rumo a uma parentalidade positiva e à vigilância do desenvolvimento infantil. Assim, também retrata pouco alcance para incrementar os ODS 3 e 5.

No tema “Caderneta da Criança: facilidades e barreiras”, há percepções que este documento é visto tanto como uma fonte rica de informações quanto barreira de entendimento. Há relatos que apontam conteúdos confiáveis e direcionados ao cuidado das crianças.

*São bem fáceis para ler e entender o que está escrito. Tem desenho também informando* [...] *é um ótimo meio de informação.* (M24 C24, 8 meses)

*Cada hora, principalmente a internet, está falando algo de diferente. Deixa a gente sem rumo, perdido para onde seguir e o que fazer. Costumo tirar muita dúvida na Caderneta da Criança.* (M15 C15, 11 meses)

Entretanto, o entendimento das informações presentes na Caderneta da Criança gera algumas dificuldades, despertando dúvidas.

*A parte da cabeça e do desenvolvimento, ainda fico confusa.* (M3 C3, 10 meses)

*Tem algumas partes que me confundo um pouco, principalmente quando falam sobre o desenvolvimento, porque nas consultas nunca é perguntado sobre esse tal de saltos do desenvolvimento.* (M5 C5, 11 meses)

Os relatos de mães que referiram o pouco uso da caderneta expressaram baixo interesse e a necessidade de maior diálogo sobre o conteúdo.

*Já folheei algumas vezes, mas ler mesmo, não. Não me chamou tanto a atenção. Gosto só de ver os dados dela.* (M26 C26, 4 meses)

*Li quando ela nasceu. Estava muito insegura em relação aos cuidados ao recém-nascido* [...] *foi bem esclarecedor. Mas falta apoio ainda mais para mães de primeira viagem.* (M6 C6, 4 meses)

O tema sobre a Caderneta da Criança revela uma comunicação profissional imprecisa, principalmente relacionada ao desenvolvimento infantil, sugerindo o pouco uso durante as consultas. Tais aspectos mostram pouca inserção dessa ferramenta na prática da puericultura na APS, a qual poderia contribuir para alcance da boa saúde no ODS 3, educação parental para o ODS 5 e promoção do desenvolvimento infantil para o ODS 10.

O tema “Busca de informações online sobre o cuidado da criança” traz relatos sobre recorrer à internet para obter informações quanto às demandas infantis.

*No Google^®^, na página de busca, procuro sobre comportamento, porque tenho um irmão que é autista, aí sempre fico ligada nesses assuntos. Também vejo muito sobre o desenvolvimento no geral, como saltos de desenvolvimento, quais comportamentos ela precisa apresentar.* (M9 C9, 5 meses)

*Eu procuro muito no aplicativo Baby Center^®^. Sigo também médicos, mas a maioria das vezes é o aplicativo de celular.* (M4 C4, 8 meses)

*Em redes sociais, gosto de seguir páginas de pediatras, de hospitais. Procuro sobre o salto do desenvolvimento para gente ver os sinais que a criança faz, o que podemos fazer para acalmar.* (M1 C1, 10 meses)

Referente às buscas diversificadas, muitos dos assuntos constam na Caderneta da Criança, mas não há uma conexão com essa ferramenta, fazendo com que os conteúdos *online* despertem mais interesse. Esses aspectos são preocupantes pela tentativa de preencher lacunas de conhecimentos sobre saúde e desenvolvimento infantil, podendo conter desinformações e estar distante da boa saúde, da promoção do desenvolvimento infantil e das práticas parentais adequadas, distanciando-se do alcance dos ODS 3, 5 e 10.

No tema “A importância de conhecer o cotidiano da criança”, é ressaltada a necessidade de abordar mais sobre as condições da família e a rotina da criança.

*Ter conhecimento do nosso dia a dia para ajudar também nos problemas, ver onde pode ser melhorado e, assim, todos sairmos ganhando.* (M3 C3, 10 meses)

*Conhecer para ajudar a cuidar bem das crianças, ainda mais as menores de 1 ano, que são tão frágeis e precisam tanto de cuidados, de atenção.* (M24 C24, 8 meses)

*Eu acho importante que saibam como é o dia dela* [...]*, se preciso conversar mais com ela, se ela está desenvolvendo bem.* (M18 C18, 9 meses)

O cotidiano da criança mais detalhado é sinalizado como importante para obter mais informações que favoreçam o bom cuidado da criança e fortaleçam as práticas parentais positivas. Nesse sentido, apreende-se que os relatos maternos apontam fragilidades assistenciais, sugerindo desafios para atingir os ODS 3, 5 e 10.

A partir dos relatos apreendidos e sua relação com os ODS, destaca-se um conjunto de fortalezas e fragilidades acerca do acompanhamento da saúde e vigilância do desenvolvimento infantil, os quais estão apresentados no Quadro 1.

**Quadro 1:** Aspectos do acompanhamento da saúde e desenvolvimento infantil na perspectiva dos Objetivos de Desenvolvimento Sustentável 3, 5 e 10

Objetivos de Desenvolvimento Sustentável	Acompanhamento da saúde e desenvolvimento infantil	
	Fortalezas	Fragilidades
Objetivo de Desenvolvimento Sustentável 3 Boa saúde	- Perguntar e conversar de vários assuntos; - Ser profissional bem atento; - Acompanhar para ver caminhos certos; - Possuir condutas adequadas; - Usar a Caderneta da Criança como fonte de informações; - Medir e avaliar em todas as consultas; - Encaixar consulta conforme necessidade.	- Dificuldades de acesso; - Rapidez das consultas; - Avaliação clínica insuficiente; - Poucas observações; - Descumprimento do calendário mínimo de consultas; - Ausência de visitas domiciliares recorrentes; - Falta de verificação sobre as fontes acessadas para obter informações infantis.
Objetivo de Desenvolvimento Sustentável 5 Empoderamento das mulheres	- Obter informação nas consultas; - Ter contato frequente com agente comunitário de saúde pelo *WhatsApp* ^®^; - Buscar ativamente informações sobre a saúde infantil; - Usar a Caderneta da Criança para obter informações; - Possuir Caderneta da Criança bem ilustrada e com dados da criança; - Conhecer a rotina da criança.	- Restrição de perguntas; - Pouca paciência; - Busca irrestrita de informações em dispositivos eletrônicos; - Pouca leitura da Caderneta da Criança; - Pouco apoio às mães primíparas; - Comunicação por mensagens restritas a avisos e lembretes.
Objetivo de Desenvolvimento Sustentável 10 Igualdade na promoção do desenvolvimento infantil	- Conhecer os problemas e desafios familiares; - Identificar o que pode ser melhorado; - Descrever estímulos e brincadeiras necessários; - Reconhecer benefícios do cuidado atento e responsivo às crianças.	- Falta de esclarecimentos sobre desenvolvimento infantil nas consultas; - Dificuldades para entender os marcos do desenvolvimento infantil; - Pouca valorização da rotina da criança e das condições familiares.

## DISCUSSÃO

Os resultados trazem expressivos apontamentos quanto ao acompanhamento da saúde da criança durante as consultas de puericultura na APS, sugerindo a necessidade de melhorar a comunicação com os cuidadores parentais, a avaliação e o monitoramento do desenvolvimento infantil. Nesse âmbito, destaca-se a relevância de avançar na prática clínica, na comunicação ampliada com as famílias e na abordagem positiva e responsiva dos profissionais de saúde para uma primeira infância saudável e para o alcance dos ODS 3, 5 e 10.

Os relatos maternos foram relacionados a um apelo para estabelecer diálogo, encadear perguntas, esclarecer dúvidas, observar e realizar exames clínicos mais completos. Houve relatos apontando que, nas consultas realizadas, é pouco conversado sobre o desenvolvimento infantil, sugerindo uma invisibilidade desse cuidado para efetivar a vigilância contínua da criança. De certo modo, são pontos que precisam avançar para proporcionar boa interação e expandir o interesse parental, que podem fornecer autoconfiança e adesão ao acompanhamento da saúde infantil. Esses elementos qualitativos estão imbricados aos ODS 3 e 5, porque tratam da boa saúde da criança e de ampliar as possibilidades de empoderamento materno para um cuidado integral, equânime e acessível.

A literatura científica traz que o acompanhamento da saúde e a vigilância do desenvolvimento infantil, de modo contínuo, apresentam resultados importantes na avaliação do desenvolvimento infantil e no aporte de conhecimentos parentais, contribuindo para identificar riscos para a saúde infantil^(28)^. O acompanhamento contínuo também contribui para prevenção de lesões não intencionais e para triagem de atraso de linguagem e do transtorno do espectro autista^(29)^.

No presente estudo, as percepções maternas denotam carência de orientações sobre estímulo ao desenvolvimento infantil, organização da rotina de cuidados e de práticas parentais pela equipe de acompanhamento da ESF, cujo enfoque dos atendimentos sugere estar na saúde física e fisiológica da criança. Estudo refere que famílias com crianças pequenas necessitam de abordagens aprofundadas sobre a qualidade das práticas parentais responsivas^(30)^. Em outros estudos, destaca-se o diálogo com as famílias pela sua importância no apoio ao crescimento e desenvolvimento saudáveis das crianças, sendo um mecanismo poderoso para estimular a ação e a responsabilização pelo desenvolvimento infantil a nível familiar e comunitário^(8,31)^.

O acesso às informações via Caderneta da Criança chamou a atenção pelo fato de algumas mães mencionarem não conseguir entender as informações que lá constam. Tais aspectos sugerem desafios do letramento em saúde e, no âmbito do presente estudo, estão relacionados principalmente às dificuldades de entendimento dos marcos do desenvolvimento infantil e às incertezas se as práticas domiciliares estariam em um bom caminho de cuidados ofertados às crianças. Estudo científico aponta que, na realidade brasileira, vêm ocorrendo distribuição insuficiente da Caderneta da Criança, poucas orientações dos profissionais às famílias, pouca participação das famílias para o uso dessa ferramenta, além de registros da vigilância do desenvolvimento infantil insuficientes e irregulares^(32)^. Portanto, são resultados importantes para os ODS 3, 5 e 10, em suas peculiaridades para a boa saúde, educação parental e promoção do desenvolvimento pleno das crianças.

As buscas em dispositivos eletrônicos, com o uso de aplicativos de celular e redes sociais *online*, trazem consonância com a contemporaneidade, porque há grande difusão do uso da Internet, sugerindo-se a obtenção de conhecimentos para suprir demandas e conteúdo de como cuidar das crianças. Por outro lado, a busca de informações em outras fontes sugere uma carência, apontando um desafio para incrementar o fortalecimento da parentalidade segura e empoderamento materno. Estudos apontam que é de suma importância que as informações digitais cada vez mais acessadas pelos cuidadores parentais sejam corretas, compreensíveis e abordem as suas preocupações^(33,34)^. Os aspectos apresentados estão particularmente relacionados ao ODS 5, tendo em vista a relevância da garantia da educação parental e das informações pertinentes ao cuidado integral em saúde. Por isso, é fundamental o acesso qualificado aos serviços de saúde e à educação em saúde para alcance de melhores resultados na APS^(18,35)^.

Os resultados também trazem sinalização sobre a rotina da criança, com percepções maternas de que é importante compreender atentamente os cuidados cotidianos, permitindo intervir e ajustar as práticas para prevenir possíveis entraves no crescimento e desenvolvimento. O processo de conhecer o cotidiano da criança e identificar os dilemas que cuidadores parentais enfrentam aparece como um valor ímpar de cuidado. Esse aspecto é corroborado pela literatura científica, que traz a relevância de um olhar atento a padrões socioeconômicos, características familiares, dos cuidadores e da parentalidade, para suprimir riscos ao desenvolvimento infantil^(36)^. Não atingir potenciais de desenvolvimento humano contribui para ciclos globais de pobreza, desigualdade e exclusão social^(22)^.

O reconhecimento da realidade diária aponta para a necessidade de se buscar, por meio do ODS 10, a redução das desigualdades de oportunidades e dos desfechos desfavoráveis às crianças. Portanto, é fundamental promover uma acuidade rica e crítica junto aos cuidadores parentais, ou seja, os profissionais de saúde desenvolverem grande capacidade de percepção da rotina de cuidados diante de diferentes circunstâncias. Pesquisadoras propõem a implementação de um mapa diário na coleta de informações que enfatizem as situações ao longo do dia com mais detalhes sobre alimentação, sono, brincadeiras, interação parental, frequência de ida à creche, dificuldades sociais, entre outras, nos períodos da manhã, tarde e noite^(37)^. Trata-se de obtenção da rotina da criança de modo mais nítido para apreender vulnerabilidades e iluminar a compreensão mais profunda das necessidades da criança no contexto familiar^(37)^.

Estudos apontam que os cuidados primários de saúde infantil representam uma oportunidade de conhecer as crianças e seus familiares em seu contexto, identificando as condições adversas que comprometem sua saúde^(38)^ e intervindo nos primeiros 1.000 dias de vida, período crucial para a melhoria da saúde infantil^(39)^. Além disso, destacam a importância de ações que articulam a vigilância simultânea do desenvolvimento e do crescimento, considerando a contextualização das situações de pobreza, que podem criar barreiras à saúde relacional e às práticas parentais cotidianas^(40)^.

Os cuidados da criança estabelecidos entre os cuidadores parentais e os serviços da APS se mostram cada vez mais importantes. Entretanto, muitos desafios estão colocados no alcance dos ODS, diante de amplos problemas globais^(41,42)^. Portanto, há desafios para investir na educação para impacto positivo na vida das mulheres, famílias, suas comunidades e, de modo mais geral, na sociedade como um todo, apontando a relação entre gênero e saúde, somando ações colaborativas entre os ODS^(43)^. Especialistas brasileiros destacam a necessidade de empenho na redução da pobreza, universalização da APS e literacia em saúde da população^(44)^ como medidas de promoção do desenvolvimento inclusivo^(45)^. As desigualdades na saúde entre os estados e regiões brasileiros afetam negativamente a população, o que pode contribuir para que o Brasil não atinja as metas dos ODS em 2030^(46)^.

### Limitações do estudo

As limitações do estudo se referem à realização das entrevistas apenas com as mães e foco no primeiro ano de vida das crianças, centradas em especificidades da realidade de um município de pequeno porte. Todavia, ressalta-se a relevância dos resultados e sua análise com base nos ODS estudados, retratando potencialidades e vulnerabilidades do acompanhamento da saúde infantil e na vigilância do desenvolvimento infantil, sugerindo formas de perceber e conduzir os cuidados de saúde na APS, que podem ter aplicabilidade no contexto das unidades com ESF, devido às suas diretrizes de proximidade às famílias e atuação no território. Outras investigações são importantes a respeito das percepções de outros cuidadores parentais e não parentais, outras faixas etárias e adversidades na infância.

### Contribuições para áreas da enfermagem e saúde

Nos cuidados primários de saúde da criança, há desafios para a enfermagem e demais profissionais de saúde para incrementar e qualificar o acompanhamento da saúde e desenvolvimento infantil relacionados aos ODS 3, 5 e 10. Assim, são necessários esforços para incrementar a interação com as mães e cuidadores parentais no acompanhamento da saúde infantil, aprofundar as comunicações por mensagens, ampliar o uso da Caderneta da Criança, compreender as buscas de informações em outras fontes, evitando a desinformação, e aperfeiçoar a obtenção de dados sobre o cotidiano da criança e sua família. Portanto, chama a atenção a necessidade de maior responsividade profissional para melhorias no cuidado das crianças e na vigilância do desenvolvimento infantil na APS para contribuir de modo mais significativo para os ODS aqui destacados.

## CONSIDERAÇÕES FINAIS

As percepções maternas trazem fortalezas e fragilidades relativas ao acompanhamento da saúde e vigilância do desenvolvimento infantil, destacando desafios para o cuidado integral das crianças e para políticas públicas. Os resultados envolvendo boa interação e comunicação efetiva com as mães, fornecimento de informações adequadas e entendimento sobre o cotidiano das famílias se alinham aos ODS estudados. Por outro lado, a rapidez de atendimentos, avaliação clínica insuficiente, comunicação frágil, baixo uso da Caderneta da Criança, uso irrestrito de informações *online* e pouca valorização da rotina e cotidiano das crianças e famílias sugerem distanciamento dos ODS analisados.

As implicações para a prática profissional se relacionam à necessidade de avanços na prática clínica, comunicação ampliada e abordagem responsiva na primeira infância. As perspectivas dos ODS 3, 5 e 10 são importantes para incrementar o acompanhamento longitudinal e integral das crianças na APS, fortalecendo a continuidade do cuidado, reforçando a premência de qualificar a comunicação e a obtenção de informações confiáveis, e lidando com as demandas diárias, particularmente para uma efetiva vigilância do desenvolvimento desde tenra idade.

## Data Availability

Os dados de pesquisa estão disponíveis no corpo do artigo.
